# Care providers’ perceptions towards challenges and opportunities for service improvement at diabetes management clinics in public primary health care in Muscat, Oman: a qualitative study

**DOI:** 10.1186/s12913-019-3866-y

**Published:** 2019-01-08

**Authors:** Kamila Al-Alawi, Ahmed Al Mandhari, Helene Johansson

**Affiliations:** 1 Department of Public Health and Clinical Medicine, Epidemiology and Global Health, Umea University, SE-90185 Umea, Sweden; 2 Department of Training and Studies, Royal Hospital, Ministry of Health, Muscat, Oman; 3 Department of Family Medicine and Public Health, Sultan Qaboos University Hospital, Muscat, Oman

**Keywords:** Oman, Primary health care, Health service challenges, Type 2 diabetes, Health care providers

## Abstract

**Background:**

The literature has described several challenges related to the quality of diabetes management clinics in public primary health care centres in Oman. These clinics continue to face challenges due to the continuous growth of individuals diagnosed with type 2 diabetes. We sought to explore the challenges faced in these clinics and discuss opportunities for improvement in Oman.

**Methods:**

This qualitative study was designed to include non-participant observations of diabetic patients and care providers during service provision at diabetes management clinics, as well as semi-structured interviews with care providers, at five purposively selected public primary health care centres. Care providers included physicians, nurses, dieticians, health educators, pharmacists, an assistant pharmacist, a psychologist, and a medical orderly. The data were analysed using qualitative content analysis.

**Results:**

The study disclosed three different models of service delivery at diabetes management clinics, which, to varying degrees, face challenges related to health centre infrastructure, technical and pharmaceutical support, and care providers’ interests, knowledge, and skills. Challenges related to the community were also found in terms of cultural beliefs, traditions, health awareness, and public transportation.

**Conclusion:**

The challenges encountered in diabetes management clinics fall within two contexts: health care centres and community. Although many challenges exist, opportunities for improvement are available. However, improvements in the quality of diabetic clinics in primary health care centres might take time and require extensive involvement, shared responsibilities, and implications from the government, health care centres, and community.

## Background

Globally, primary health care practice organizes and provides care for both acutely and chronically ill patients [1]. The primary care system elicits and reviews data concerning the course and management of patient’s diseases. It 1) helps patients to set goals and solve problems for improved self-management; 2) provides clinical and behavioural interventions that prevent complications and optimize disease control and patient well-being, and 3) ensures continuous follow-up [1].

Primary health care is the first line of care provision and the most accessible service for the population [2, 3]. Despite its easy accessibility, primary health care services globally, and in the Arab world specifically, still face numerous challenges, such as inadequate implementation of evidence-based medicine, challenges related to teamwork, impaired information systems, and challenges related to ethics (e.g., care providers’ lack of knowledge and skills) [4–6]. In Arab countries, the government expends considerable effort to overcome these challenges, improve services, and provide high-quality care for their patients [7], as easy accessibility and effective health care services are considered key components of optimal health care [8]. Therefore, it is very important to address the different challenges and discuss various opportunities to achieve these goals.

Diabetes management is given special attention at the primary health care level because of the disease’s epidemic nature and the fact that its prevalence is continuously increasing [9]. Globally, over the past three decades the number of people with type 2 diabetes has more than doubled, making it one of the most important public health challenges [10, 11]. The situation in Arab countries is not different. Many of the countries with the highest prevalence of type 2 diabetes in the world are in the Arab world [12]. In Oman, for example, the burden of type 2 diabetes has increased among adults aged 20 years and older rising from 8.3% in 1991 to 11.6% in 2000 [13]. The World Health Organization has predicted a 190% increase in the number of people living with type 2 diabetes in Oman from 75,000 in 2000 to 217,000 in 2025 [13].

Therefore, it is important to explore primary care and diabetes management care provision in Oman, recognize any challenges, and address the various opportunities for improved quality of care at different levels [14–16]. The literature already recognizes and discusses several challenges in diabetes management clinics at the primary care level in Oman, including health care providers’ communication skills, doctors’ and nurses’ performance, and patients’ service dissatisfaction [17–19]. Furthermore, the public diabetes management clinics, including the care providers within the core diabetes management team (physician, nurse, dietician, and health educator), face challenges with diabetes service delivery in terms of the team-based approach and the distribution of resources, while the national guidelines require proficient application for patients to receive the best quality of care [20]. Diabetes management at the primary care level continues to face challenges, both within health care centres and in the community, such as patient self-management and support, all of which require prioritized actions with appropriate solutions. Therefore, at this stage, it is important to identify all of the different challenges related to the diabetes management clinics at primary care centres and discuss opportunities for improvement to raise the level of primary health care diabetes management services [21].

## Methods

### Study aim

This study aimed to explore challenges and discuss opportunities for improvement of diabetes management clinics in primary health care centres in Oman.

### Study design

This study is part of a bigger project aimed at exploring the feasibility of interdisciplinary teams in the management of diabetes at the primary health care level in Oman. This study took a qualitative approach and was designed to include non-participant observations of diabetic patients and care providers during diabetic service provision in addition to semi-structured interviews with care providers involved in these clinics to identify challenges and discuss opportunities for improvement.

### Study setting

The study was conducted in Muscat, the capital city of Oman, which has a population of 4.6 million [22]. Muscat is divided into six provinces and 50% of the total population lives there. The ethnicity of Muscat’s comprises a mix of Omanis and non-Omani citizens from various countries, including those of Arab, South Asian (Pakistani, Sri Lankan, Bangladeshi), European and African origin. The official language is Arabic though English, Balochi, Urdu and Indian dialects are also spoken [23]. The varied population of Muscat is served through different public and private health sectors, which include primary, secondary, and tertiary care services. Omani citizens can use both public and private health sector services whereas non-Omani citizens predominantly use the private sector care. The Ministry of Health is the main body that covers health care expenses, which consume around 3.5% of the country’s total GDP [24]. On average, healthcare centres cater for approximately 90% of the population’s needs [25]. The primary care services involve general physicians, specialized family practitioners, nurses, dieticians, and health educators, in addition to other support staff. The secondary and tertiary health care services involve more specialized health care providers. At all three levels of services, the level of care management fluctuates according to the severity of the patients’ disease and is provided free of charge. The management includes consultations, laboratory tests, invasive/non-invasive procedures, dispensing medication, and follow-up appointments. Within the public primary health care sector in Muscat, which comprises 27 centres, 26 provide diabetes management services.

#### Public primary health care centres

##### Standard structure

A standard public primary care centre in Oman consists of a reception, eight consultation rooms, separate waiting areas for men and women, an administration room, a dietician’s and a health educator’s room, a treatment room (which receives patients for short observation and minor emergencies), a radiology room, a small laboratory, a pharmacy, and a room for security staff and an ambulance.

##### Regular staff

In general, the permanent staff that are available in the centre include physicians, nurses, a dietician, a health educator, a radiographer, a psychologist (not in every centre), pharmacists, assistant pharmacists, lab technicians, medical orderlies (non-medical staff who are responsible for handling the service flow at the centre), an ambulance driver, records’ clerks, cleaners, and security staff. In addition to the permanent staff, other temporary medical students and residents are available; these individuals are appointed in the centre according to their medical rotations. The main nationality of physicians is Omani, although other physicians are recruited from different Asian and African countries. The other providers are mainly Omanis, with only few Indian nurses. All support staff are Omanis.

##### Regular diabetes management clinics

The regular diabetes management clinics in public health centres are usually conducted according to national diabetes guidelines and regulated by four team members (a physician, nurse, dietician, and health educator) [20]. These clinics are combined with hypertension clinics and are delivered in the mornings (further characteristics of the regular diabetes management clinic are described in the results section).

### Study sampling

#### Health care centres

The selection of health care centres for this study aimed at maximum variation. Based on the information collected from the first study in the project [20], and the national diabetes guidelines in place at the time of this study, five centres were selected. These centres included: a) an old/non-standard structured primary health care centre with mixed nationalities of care providers on the diabetes management team; b) a centre of standard structure with only Omani care providers on the diabetes management team; c) a centre of standard structure with a lack of pharmaceutical and technical resources related to diabetes management team; d) a centre of standard structure which lacked some of the regular care providers from the diabetes management team; and e) a centre of standard structure with the highest number of diabetic patients recorded per centre and the presence of additional care providers as well as the standard diabetes management team.

#### Care providers

All available care providers who served in diabetes management clinics in the selected centres at the time of the study were included. They included: physicians (Omani and non-Omani), nurses (Omani), dieticians, health educators, pharmacists, an assistant pharmacist, a psychologist, and a medical orderly (Omani).

#### Patients

All patients in the centres’ registration system (daily appointment list for diabetes management clinic) aged ≥19 years old and had type 2 diabetes were included in the study.

### Data collection

Data collection started in January 2016 and ended in March 2016. One week was appointed for each of the five centres to complete data collection (from Sunday to Thursday). The centres were visited every morning and a further visit was made in the afternoon if necessary. The primary centres were visited on more than one occasion to capture as much information related to the study as possible and to minimize the Hawthorn effect on the care providers side’ [26] (i.e., the same care providers were observed during several consultations for more than 1 day). The patients’ list for the diabetes management clinic was requested from the centre’s registration system on every day of the clinic and the formalities for the interviews were set on a daily basis between the researcher, the physician in charge, and the participants.

#### Observations

The main aim of the observations was to better understand the diabetes management clinic and its service provision, so that it could be used as a basis for the interviews. A protocol was developed in advance by the research team and was based on national guidelines and data collected for the first study in the project [20]. The protocol involved collecting two sets of data. The first set involved collecting provider and patient characteristics. The second part involved recording observations about a list of items related to the provider’s tasks and roles. The items included on this list were: performance of different tasks/responsibilities, performance of management tasks and performance of external transfers/referrals, if required. The first author observed the diabetic patients when they entered the first consultation room where a physician (alone or with a nurse) was located, and then followed them to another consultation room if they were referred to see other members of the team. The first author also attended meetings conducted between the staff where the patients’ cases were discussed. There were no refusals to be observed from either the care providers or patients.

#### Interviews

The research team developed the interview protocol for the care providers with open-ended questions, and the first author conducted the interviews. The questions were related to the organization of work in the diabetes management clinic, the informal mechanisms for work organization, the relationship between the health care providers and their professional role. The main focus was to identify different problems/challenges that might occur within the current work organization or between the professionals and discuss opportunities for improvement. The participants were individually interviewed for 30–45 min at the end of the clinic service. The interviews were conducted in English or Arabic, audio recorded, transcribed verbatim, translated to English when necessary, and stored securely. Personal details were removed and stored separately. One provider was not able to participate due to time constraints.

Table 1 displays the participants and their different characteristics. The ideal plan was to observe the patient during care provision with all members of the team and subsequently interview all care providers involved. However, due to time constraints and the crowdedness of the clinics this could not be achieved. Therefore, 14 patients were observed, four care providers were only observed, 18 care providers were only interviewed, and nine care providers were observed and then interviewed. The majority of care providers were female. Only two males were involved; one was observed, and the other was interviewed. Similarly, the majority of observed patients were female; only one male was observed.

**Table 1:** Characteristics of study participants

Participants	Observed only			Interviewed only					Observed and then interviewed				
Profession	P	N	D	P	N	D	HE	O	P	N	D	HE	O
Female	O	2	1	7	6	1	1	2	3	2	1	1	2
Male	1	0	0	1	0	0	0	0	0	0	0	0	0
Age, years	30–45			30–45					30–45				
Experience, years	5–20			5–20					5–20				
Nationality	Egyptian, Indian, Omani			Omani, Sudanese					Omani				
Language	English and Arabic			English and Arabic					English and Arabic				
Patients													
Sex													
Female	13			–					–				
Male	1			–					–				
Age, years	30–80 years												
Duration of type 2 diabetes, years	5–20 years												
Nationality	Omani												

*P* Physicians, *N* Nurses, *D* Dieticians, *HE* Health Educators, *O* Others (pharmacist, assistant pharmacist, psychologist and medical Orderly)

### Data analysis

#### Observations

The observations were manually analysed into descriptive summaries. The summaries describe three different models of service delivery at diabetes management clinics and three visit types (first visit, regular follow-up visit, and annual visit). The care providers’ tasks and roles were included in the summaries.

#### Interviews

Data were analysed manually using qualitative manifest content analysis. Manifest content analysis was based on close statements of the care providers’ words without extensions to the underlying meanings [27]. The transcriptions were read through several times to ensure familiarisation with the data. Text dealing with challenges related to diabetes management clinics and the implications of these challenges (outcomes) were identified as meaning units, which were condensed, interpreted, and labelled with codes. The identified challenges with similar content were developed further into sub-categories and categories, and the implications of the challenges were listed as groups for each sub-category. Finally, the categories were further grouped into one of two contexts.

The actual coding was performed by the first author, but was discussed in regular peer-debriefing sessions to enhance the trustworthiness of the findings.

### Ethical considerations

The Research and Ethical Review and Approve Committee in Oman approved the total project, including the current study. The participants were verbally informed about the study. Their participation was clearly explained and was entirely based on voluntary contribution, and the collected information was handled confidentially. Verbal consent was obtained from every observed participant, and additional written consent was obtained from every participant who agreed to be interviewed.

## Results

The result section starts by outlining the three different models of service delivery at diabetes management clinics, including the care provider’ tasks and roles that arose from the observations. Then, the section leads the reader to general and specific challenges related to the different models of service delivery of diabetes management clinics and community that were discussed by the care providers.

### Different models of service delivery at diabetes management clinics

#### First deliver model - a regular combined morning clinic for diabetic and hypertensive patients

This clinic is the regular diabetes management clinic and was available in four of the five selected centres. The clinic provided a combined service for diabetic and hypertensive patients. The clinic was conducted four to five days per week from 8 am to 2 pm and was mainly led by a physician and a nurse in the same room. The room was 15 m^2^ and contained two desks: one for the physician and one for the nurse. The clinic had one computer on the physician’s desk, manual diabetes registries on the nurse’s desk, and various tools for clinical examination of patients including tools to collect weight/height/waist circumference measurements, a finger prick machine for blood sugar testing, diabetic foot examination tools, and a single bed for patients’ full body examinations. The clinic received an average of 8–10 registered diabetic patients, 6–8 registered hypertensive patients per day, and 2–4 patients without appointments as emergency visits. When required, patients were referred to other members of the team (including the dietician and the health educator) who were located in the same centre in a room of 10 m^2^. The patients attended the clinic according to an appointment system and according to their visit type: first-time visit, follow-up visit (every 3 months), or annual visit. During the first-time visit, a nurse was the first care provider who welcomed the patient, registered him/her as a diabetic patient (usually referred from a general clinic in the same centre), and provided him/her with a logbook for sugar measurements. Then, the nurse took patients’ weight/height/waist circumference, blood pressure, glucose reading (finger prick), and examined their feet. In the same room, she asked the patient to see the physician. The physician’s role was to take the patients’ complete medical history, perform a physical examination (general and cardiovascular), prescribe suitable medications (drugs/insulin), request complete laboratory investigations related to diabetes and an electrocardiogram (if not ordered by the general practitioner), and refer the patient to the dietician and health educator. The dietician was responsible for collecting a complete dietary history, including traditions and daily exercises, and providing a complete healthy dietary and exercise plan. The health educator was responsible for discussing the prevention plan, including any related social issues. The encounter between the patient, the nurse, and the physician took 15 to 20 min and the encounter between the patient, the dietician, and the health educator took 10 min. Follow-up visits were usually shorter (10 to 15 min). In follow-up visits, the nurse performed the same tasks; however, the physician only asked about the patient’s complaints, reviewed medications, and referred the patient to the rest of the team if required. The annual visit was performed after patients completed 1 year in the diabetes management clinic. During the annual visit, the nurse performed the same tasks as in previous visits. The physician completed the same tasks as in the follow-up visit, repeated all laboratory investigations and the electrocardiogram, and referred the patient to the dietician and health educator in the same centre and to an ophthalmologist at a secondary care centre. The annual visit lasted for 15 to 20 min. After completion of the consultation with the physician, the nurse, the dietician, and the health educator, the patient approached the pharmacy for medication dispensing and the registration desk to book the next appointment. No organized team discussions were observed.

#### Second deliver model - an additional afternoon clinic for sole diabetes management

This type of diabetes management clinic was available in two of the four centres where the regular combined morning clinic was already present. The purpose of opening the additional exclusive diabetes afternoon clinic was to devote more time during the service to chronic patients who had uncontrolled and complicated diabetes as well as patients on insulin. The service was provided twice a month from 3 pm to 7 pm and was conducted by a team composed of a physician, a nurse, a pharmacist/assistant pharmacist, a dietician, a health educator, and a medical orderly. This clinic utilized a waiting area and four consultation rooms, in which each member of the team consulted the patient individually. This clinic was not available in every centre due to the shortage of staff (as revealed in the interviews). The patient list contained eight to 10 patients per clinic per day, and the service was provided on a forward/backward system. This means that patients were transferred to this clinic to bring their disease under control and then shifted back to the morning clinic after the disease had settled. The first-time visit was usually not conducted in this setting. The clinic started with a meeting between the staff, which included a review of the cases for that day. Then, the medical orderly staff called the patients to confirm their appointments. After, the medical orderly staff welcomed patients on arrival and directed them to a triage room where a nurse and medical orderly were situated. The medical orderly’s role was to register the patient, and the nurse’s role was to take the vital signs (weight/height/waist circumference), blood glucose (finger prick) and blood pressure measurements. Then, the patient was welcomed to another room to see the physician who asked about the patient’s complaints and discussed the treatment plan in detail. All laboratory tests were conducted during the morning visit before the patient was moved into the afternoon clinic and was checked by the physician to determine if any follow-up was needed. After the physician’s consultation, a pharmacist in a different room welcomed the patient. The pharmacist reviewed the complete list of medications, including side effects and storage. Because this clinic was mainly for patients with complications and those on insulin, the consultation times with the physician and the pharmacist took up to 15 min each. The final station for the patient was with the dietician and health educator who reviewed dietary habits, physical exercise plans, and healthy lifestyles and discussed any social challenges related to or affected by the patient’s compliance. On some occasions during the afternoon, the dietician and the health educator provided their diabetic services in community settings and thus could not attend the afternoon clinic. In addition, on some occasions the patient was sent to a psychologist for an individual appointment. The patient’s total stay at the clinic took 14 to 15 min per visit for either a follow-up visit or an annual visit. After the service was completed, the health care providers held a second meeting to discuss the patient list again and decide who should be transferred to the morning clinic, who should be continued in the afternoon clinic, what the patients main concerns were, and which of the team members would benefit them more. The meeting ended with several decisions and further actions to be performed.

#### Third deliver model - a general clinic

This type of clinic was a general clinic, conducted not only for diabetic patients but also for other patients who visited the centre on that day. The complete diabetes management team was not available and, subsequently, the service was provided using a single physician approach. This clinic was available in one of the five selected centres. The first provider was nurse, who welcomed the patient and measured his or her vital signs (weight/height), blood glucose (finger prick) and blood pressure measurements. Then, the physician conducted the consultation, which included taking the patient’s history, performing a complete physical examination, drawing blood samples, ordering laboratory investigations, prescribing medication, and discussing a complete plan of dietary tasks and physical exercises to ensure that the patient received a complete healthy lifestyle package. All three types of visit (first, follow-up, and annual) involved the same consultation approach and lasted for 15 to 20 min.

#### Challenges related to diabetes management clinics

The analysis of the interviews with care providers resulted in capturing general and specific challenges related to diabetes management clinics in primary care and consequent challenges related to the community. The challenges are divided into two different contexts: health care centre and community. The findings, including the implications of the challenges (outcomes), are summarized in Table 2. The presentation of results follows the order set out in the table and direct quotations from the interviews are included to illustrate how the interpretation is grounded in the data.

A) Health care centre

1. *Infrastructure.*
  - Shared rooms.
    The discussion with the care providers revealed that shared rooms in clinics were common practice. The health centre contained several shared room clinics, including the diabetes management clinic (in the regular combined morning clinic), the asthma clinic, and the antenatal clinic. These shared room clinics required two providers to sit together. Commonly, the physician and the nurse sit together, and the dietician and the health educator sit together. The practice was considered a challenge for all the providers involved. The providers revealed that to work efficiently, each provider should have his/her own space, tasks, and responsibilities. Otherwise, many unintended consequences might occur for the provider, including seeing one patient at a time per room while the other provider is waiting, or not having the chance to independently interview the patient, which would consequently lead to a waste of providers and patients time. Therefore, it was suggested by the care providers that new centres should be built with additional rooms.
*‘The nurse can do a lot, but because the place is not big enough she can’t, but if she had a separate place, she could interview, check patients’ compliance, and take more history from the patient.’ (Physician, Omani, First model of service delivery)*
*‘I prefer to see the patients individually, but it is difficult when I am sharing the room with the health educator: she has to leave the room, and if she has a patient, I have to leave the room; it is a waste of time on both sides.’ (Dietician, Omani, First model of service delivery)*
  - Shortage of computers.
    Computers play important roles in any health organization, and their presence is essential for proper patient management. Therefore, health centres are expected to have several rooms with an adequate number of computers. However, the care providers admitted that in addition to shared rooms, they also had to share computers. In the combined morning diabetes management clinic, the providers revealed that one computer per two providers might lead to management difficulties and job restrictions. The nurses specifically disclosed that regular data entry is not possible. They added that because there are not enough computers, they had to stay behind after hours to finish work that they could not finish during their shifts. Loss of patients’ data was also reported. Therefore, additional computers were suggested.
*‘The doctor and nurse sit in one room with one computer, and the dietician and health educator also share a room, with one computer. Our job is restricted by this situation.’ (Physician, Non-Omani, First model of service delivery)*
*‘Sometimes I have to stay after working hours to finish entering patients’ information into the computer as during the service the doctor was using the computer. We have only one computer; if another computer were provided the restricted situation could be solved.’ (Nurse, Omani, First and second models of service delivery)*
  c) Incompetent support system (IT).
    Each health centre consists of a support system that is responsible for registration of patients, handling appointment dates, and saving statistical data. The dieticians disclosed that the registration system, which connects to the appointment system and the annual statistical data at the primary centres was not efficient. They added that the patients were affected by this, as it was not known how many visits the patients had had with the dieticians and what had been discussed with them, which indicated that an important part of the patients’ care was missing.
*‘The registration system available in the centre is not good at all, it only gives information on the number of patients seen by the doctor, and when the doctor refers a patient to me, it does not show it in the system. So my statistical data is missing.’(Dietician, Omani, First model of service delivery)*
    In addition, the physicians disclosed that the registration system at the diabetes management clinic allows 18 patients to be registered per day allocating 20 min per patient: this does not leave any empty slots for emergency patients. This method creates an overbooked list, insufficient consultation time, and no solution for following up defaulters (patients who do not turn up for their appointments), which leads to a discontinuity in care. Furthermore, the system described did not show suitable flexibility to adjust the length of appointments to the patients needs and requirements, which affected the quality of care, as it did not allow the providers to deliver individual and tailored care for each patient. Therefore, the care providers had reached out to the ministry, which is considered the main body responsible for technical solutions, to ask for system evaluation.
*‘We have around 20 minutes for each patient, but it is very difficult to follow this strictly because of the overbooking list and the absence of empty slots for emergencies, which leads to shortening the consultation time for the booked patients to allow un-booked patients to be seen.’(Physician, Omani, First model of service delivery)*
*‘The current biggest problem for us is the default problem. We do not have a proper system to trace the non-attending patients, and consequently, their process of care is discontinued with the current system: the ministry should look into this problem seriously.’(Physician, Non-Omani, First model of service delivery)*
    The psychologist in the second model specifically also revealed that there was no connection between primary and secondary health care registration systems. Therefore, it was not easy to follow-up with patients, which had serious implications for the diabetes management clinic and for the patients, who did not have a complete file of their disease management history at the site where they registered and received their treatment. A solution to this challenge was also requested from the ministry.
*‘It would be good if all three care levels were connected electronically, so I would be able to follow-up the patient, and know where s/he had gone. Now, with this system the patient’s file is incomplete: the ministry should update the system.’(Psychologist, Omani, First model of service delivery)*
  d) Shortage of providers.
    The providers in all three models of service delivery disclosed that not all members of the diabetes management team are always available during service provision in the morning (or in the afternoon where applicable). This might be due to a shortage of staff or involvement in other activities in the community. In such circumstances, when the absence of the care providers was confirmed, the physicians had to perform other providers’ roles in addition to their own. This approach consequently leads the physicians to feel tired, overstretched, and unfocused, which will affect the service and the patients. The third model of service delivery was described as a single provider’s role, and the nurses revealed that the tasks were not professionally distributed, meaning that the full team was not available, and members could not complete their tasks, because the available providers were performing the tasks of those who were missing. In the first and second models, the nurses added that even if they wanted to separate the combined clinic, it was never possible due to the shortage of care providers and that afternoon clinics sometimes needed to be cancelled for the same reason. The pharmacists revealed that staffing shortages sometimes occurred in the pharmacy, and this had serious implications for the service as they had large numbers of patients and a limited time to provide a quality service for these patients. Therefore, the care providers in all three models saw additional human resources of different professions (but mainly nurses) as a good solution.
*‘Shortage is mainly in the nurses. Sometimes the doctors run the (diabetic) clinic without the staff nurses. This put a lot of pressure on the doctors; they become tired and unfocused.’(Physician, Omani, First model of service delivery)*
*‘We have an extreme shortage of nurses. We run a combined (diabetic and hypertensive) clinic in the morning but even if we want to separate the clinics, we can’t.’(Nurse, Omani, First model of service delivery)*
*‘In our model of service delivery, doctors perform the main job; they have to do the majority of tasks because we do not have a diabetes management team, and this leads to an unprofessional distribution of tasks. Thus, we require additional professional staff.’(Nurse, Omani, Third model of service delivery)*
*‘In the morning shift, I can’t communicate with the patients properly; the time is short, there are only two of us in the pharmacy, and we have to cover a large number of patients so we work very fast because we want to finish. It ends up with low-quality service being provided to the patients.’(Pharmacist, Omani, First model of service delivery)*
  e) Non-Arabic speaking providers.
    Communication between care providers and diabetic patients plays a very important role in the management of the disease. Discussions with care providers suggested that there were several challenges related to communication within the clinic and the pharmacy, including older Arabic speaking patients being treated by non-Arabic speaking providers, which might lead to an increased number of defaulters, and consequently to disturbed continuity of care. The care providers did not provide any solution or any possibility for improvement.
*‘Our patients are quite old and do not speak English, and if they come to the clinic but do not understand the provider they do not come again, and we lose them and the continuity of care is disturbed.’(Physician, Non-Omani, Third model of service delivery)*
*‘The pharmacy staff do not speak Arabic at all, and most of our patients are old and only speak Arabic: that leads to communication problems.’(Pharmacist, Non-Omani, Third model of service delivery)*
2. Tools/technical resources.
  - Shortage of dieticians’ and health educators’ diagnostic and educational tools.
    Dieticians reported that technical resources, including educational and diagnostic tools, were not adaptive to diabetes management clinics and were not available in general in the centre. Educational tools and diagnostic tools for dieticians and health educators specifically were very limited, and this situation very often discouraged the patients from completing follow-up at the public centres. The dieticians also added that such challenges had serious implications for the diabetes management clinics, as the patients consequently go to private clinics, even if they have to pay, to receive proper management. The care providers revealed that revision of the available resources should be part of the ministry’s regular responsibilities.
*‘I do not have proper resources and educational tools that can help me in my clinic and the ministry should look into that, the patients are not encouraged to see me because of this, they prefer to go to private clinics and pay to see a dietician because s/he has everything. So it is difficult to say that the patients in our clinic are fully educated.’(Dietician, Omani, First model of service delivery)*
  - Outdated diabetic drugs and shortage of cardiovascular drugs.
    According to the primary health care physicians, medications for diabetes management clinics were not fully available, not updated, and sometimes were not effective for the patients. The care providers also revealed not only that they had limited numbers of hypoglycaemic drugs, but also that they faced a shortage of cardiovascular drugs; this requires improvements by the pharmaceutical companies, who usually are the ones responsible for providing the drugs. Such challenges lead to ineffective medical management at the public clinics and force patients to buy their medications from outside pharmacies instead of getting them free at the centres. To solve such a challenge, the care providers suggested that pharmaceutical companies should be improved to provide complete management at the centre.
*‘Medications are an issue; they are very limited for us. I have been practicing for the last 17 years as a primary health care physician; we still have the same medications that we had then. Diabetic cases have increased and we have more patients and greater incidences in younger patients, but still we have the same medications since that time. I think pharmaceutical companies in general should be improved so the medical management could be complete and our patients continue to visit us and our pharmacy instead of outside pharmacies.’(Physician, Omani, First model of service delivery)*
  Some of the physicians added that they provided some solutions for patients to get the medications required from outside pharmacies, but that they had been questioned for their actions, which provided great discomfort.
*‘I prescribed medications from outside for the patients, but I was questioned about why I was giving medications that can be bought outside.’(Physician, Omani, First model of service delivery)*
3. Interests/knowledge/skills.
  - Nurses low interest in diabetes.
    Physicians in all three settings stressed that nurses had little interest in diabetes management. The nurses were described as disinterested and unfocused, and physicians claimed that whenever they were sent to a workshop to learn a new skill, it was not applied. The physicians explained that these challenges may further lead to lost clinical skills and underutilized nurses, and suggested that with proper selection of nurses for the workshops, proper supervision from the nurses in charge, and proper follow-up, evaluation, and appreciation from higher authorities, the situation could be improved.
*‘For some people, it (diabetes) is not a fascinating subject. Still, you should train people who have an interest in the subject. I think they should select people (nurses) who have interest, not just for a promotion or selecting a fixed job. Otherwise, our nurses who are trained in diabetes will not bother to apply their skills, and these skills will be lost.’(Physician, Omani, First model of service delivery)*
    The nurses, on the other hand, revealed that they had interest and the skills in diabetes management, but that they could only apply them if the higher authorities provide legal authorization for the job. They also added that official recognition of their role as diabetic nurses could give them a chance to receive permanent jobs in the diabetic clinic instead of having to cover other clinics in the centre. Moreover, they disclosed that having a permanent job would ultimately be better for them as nurses and for the patients in terms of quality of care provided and satisfaction.
*‘It is good to have a fixed job rather than being moved every day to a different clinic. It is very difficult to focus on the work if you have to do something different every day.’(Nurse, Omani, First and second models of service delivery)*
*‘It would be a very good idea for the nurses if we are officially certified and given a name (diabetic nurse) because this will support the argument that we can do this job and later become labelled as nurses practitioners.’(Nurse, Omani, First model of service delivery)*

b) Low nurse knowledge and skills
  The majority of physicians reported serious concern about nurses knowledge and skills level in diabetes management, which meant that work was not delegated to them, resulting in nurses who were not confident in the clinics. The physicians also mentioned that they had noticed low patient trust in nurses, which led to low nurse contribution to the service and underutilized nurses. The physicians added that because of the current situation, they became distressed and overwhelmed due to the tasks that had been given to them that were not related to their job description. On the other hand, and in contrast to the physicians, the nurses did not mention their knowledge and skills as a weakness or a challenge. They confirmed that their management was appropriate, but that patients still prefer to see the doctor. They also added that the patients trust the physicians more than them; however, they feel that they can provide the service with the same quality of care.
*‘Coming back to the nurses, there are some issues. They are undertrained; they just go to certain workshops to gain skills. Some of them are not aware of simple drugs, which are used in diabetes. At least the mechanism of action in cases of hypoglycaemia should be well known. Likewise, medications that cause hypoglycaemia should be known. Health professionals should know things like these. I think they should be more utilised and better equipped and trained, and they will be of great help for us, as management of diabetes requires teamwork. Otherwise, it will be critical to delegate any work to them’.(Physician, Omani, First model of service delivery)*

**Table 2:** Contexts, categories, sub-categories and outcomes

Contexts	Categories	Sub-categories	Outcomes
A) Health care centre.	1) Infrastructure.	a) Shared rooms.	• Providers cannot interview the patient independently. • Handle one patient at a time, which leads to waste of providers’ time.
		b) Shortage of computers.	• Inefficient management. • Loss of patient data.
		c) Incompetent support system (IT).	• Missing data for annual statistics. • Inefficient appointment system. • Twenty-minutes allowance only for consultation. • Overbooking system and follow-up failure.
		d) Shortage of providers.	• Physicians have to perform other providers’ roles and manage all aspects of the illness. • Tasks are not distributed according to professional abilities, which increase the single physician approach. • Physicians are tired and not focused. • Inability for pharmacists to provide quality time for patients. • Impossible to separate the hypertension and diabetes clinic. • Cancelation of afternoon clinics.
		e) Non-Arabic speaking providers.	• Communication barrier between provider and patient. • Increased number of defaulters and consequently discontinuity of care.
	2) Tools/technical/pharmaceutical resources.	a) Shortage of dieticians’ and health educators’ diagnostic and educational tools.	• Patients not managed/educated properly at the centre. • Patients prefer to go to private clinics.
		b) Outdated diabetic drugs and a shortage of cardiovascular drugs.	• Ineffective medical management. • Patients have to purchase the drugs from outside pharmacies.
	3) Interests/knowledge/skills.	a) Nurses interest in diabetes low.	• Nurses not trained in diabetes care. • Loss of skill application in trained nurses. • Non-focused nurses due to their distribution in different clinics.
		b) Low nurse knowledge and skills.	• No delegation of work from physicians to nurses. • Low patient trust in nurses. • Underutilized nurses.
B) Community.	1) Cultural beliefs/traditions.	a) Listen to and trust friends/family more than health providers.	• Seeking traditional treatment/healers. • Seeking second opinions. • Low compliance with treatment. • Increased defaulters.
		b) Non-commitment to appointment system.	• Clinic crowdedness. • Disturbed providers.
		c) Sweet diet and sedentary lifestyle.	• Low compliance with healthy lifestyle.
	2) Knowledge/awareness.	a) Lack of health and diabetes awareness.	• Denial of diabetes. • Low compliance with medication. • Low compliance with healthy lifestyle.
	3) Transportation.	a) Lack of (public) transportation (patients dependent on family support).	• Increased defaulters. • Discontinuity of care.

*‘The patients only trust the doctors; they think that we do not know how to handle them. We have noticed that, but we know that we can handle the patients properly.’(Nurse, Omani, Third model of service delivery)*

*‘The patients only trust the doctors, and they want to see only them, even when I tell them that they will have to wait for a long time for the doctor, but the nurse is available immediately. This is very common when patients without appointments come to the clinic.’(Medical Orderly, Omani, First and second models of service delivery)*

B) *Community*

1. Cultural beliefs/traditions
  - Listen to and trust friends/family more than care providers
    Diabetes and its management through diet, drugs or insulin injections is never an easy subject to discuss in the community. Care providers revealed that people in the community have always listened more to each other than to care providers concerning diet, diabetes management, and health. Consequently, this has had distressing consequences on their health and has led to patients not attending appointments. Despite the care providers continuously providing the correct knowledge, low acceptance from the patients has been a challenge. The care providers also mentioned, that rejection of treatment modalities, going to traditional healers, and searching for second opinions are very common cultural behaviours. Such traditions lead to increases in the number of defaulters as well as low compliance with drug, insulin, and lifestyle plans.
*‘Community really has a big effect on health awareness and the relationships between the people are very strong. Diabetic patients always advise each other and recommend doctors for second opinions or help from a traditional healer.’(Physician, Omani, First and second models of service delivery)*
  - Non-commitment to the appointment system
    The nurses mentioned an important concern, which causes dissatisfaction and distress among all of the care providers in both the diabetes management clinics and other clinics in the health centre, namely overcrowding. The nurses admitted that patients disregard for the appointment system was the main reason for this overcrowding. They explained that some patients arrive very early, some arrive very late, and some come without appointments, but because all patients want to be seen immediately resulting in a stressful environment and disruption to the providers. The care providers suggested communication with the patients could improve this issue, but this could take time, and further support from the community is required.
*‘We are facing a lot of problems with patients who are not committed to the appointment system; some of them come too late and some of them too early for their appointments, and this is a big problem. It leads to serious crowding in the clinic and major disturbance to the providers.’(Nurse, Omani, Third model of service delivery)*
*‘It is not easy to solve this (non-commitment) problem because it is related to patients’ behaviour, but with communication, we can achieve a solution, although we can’t do it alone; we need help from the community.’(Nurse, Omani, First and second models of service delivery)*
  - Sweet diet and sedentary lifestyle
    The care providers described that the Omani diet includes sweet foods and sedentary lifestyle habits, which directly affects individuals’ health and specifically diabetic patients’ compliance. The health educators admitted that their patients eat a lot of food that contains large quantities of sugar and that it is difficult for them to commit to a healthy lifestyle. The dieticians also expressed their worries about the traditional Omani diet, as it is full of sugar and fat. If patient compliance could be improved, the resources in the diabetic clinics could be used more efficiently and directed to patients who need them. Solutions to these challenges were mentioned in the context of collaboration between the Ministry of Health and the media.

*‘It is very difficult for patients who have diabetes to perform healthy dietary commitments. They are more committed to the Omani food and culture.’(Health Educator, Omani, First model of service delivery)*

*‘Their (diabetic patients’) diet is full of sugar and fat, and this is due to Omani dietary traditions.’(Dietician, Omani, First and second models of service delivery)*

*‘Media can help us in this challenge (the Omani tradition of sweet food), and the government has already started to look into different ways to deliver healthy life messages to the population through television, newspapers, and magazines.’(Physician, Omani, First model of service delivery)*

2) Knowledge/awareness.
  - Lack of health and diabetes awareness
    The community knowledge that was disclosed by the care providers included health and diabetes awareness. According to the care providers, the people in the community have different levels of understanding towards health awareness and poor knowledge about diabetes. This has always led to great denial among patients about the disease itself, low compliance with medication, and rejection of healthy lifestyles.
*‘They (diabetic patients) should know how to control their diabetes, but some of them are still in denial that they have diabetes and they stop their insulin. Some of them will go for traditional or other treatment. Glucometers are given for free to each diabetic patient, so there is a problem in the community.’(Physician, Omani, First model of service delivery)*
    In addition, the physicians mentioned that many of the conditions with which patients come to the centre could be prevented at home, which would lead to saving a lot of the centre resources. For example, controlling blood sugar levels by introducing insulin injections when required and taking medications on time could prevent patients from experiencing unnecessary diabetic comas. The situation is not always ideal and leads to a waste of resources and providers’ time, energy, and concentration. However, the care providers believed that building strong awareness in the community is one of the key factors to solve this problem.
*‘If there is health awareness that is built into the community, people will know what can be done at home, and what can be done in the health centres or hospitals. Properly utilized health centres would give us the opportunity to do more; we could address more, give more and provide more time. I think we would do much better work. I think building awareness is very important.’(Physician, Omani, First model of service delivery)*

3) Transportation.
  - Unavailable (public) transportation
    The care providers revealed that Oman’s public transportation system is not yet fully developed. Therefore, patients ask family members to drive them to appointments, as most are old and not independent. They also added that this challenge is not specific to diabetic patients, as it is a concern for most patients coming to public centres. Problems with transportation represent the main reason for the high number of defaulters; patients do not turn up for their appointments, and there is no opportunity for follow-up. The situation causes increased numbers of defaulters, missed consultations, and discontinuity of care. The care providers revealed that one way to solve this problem was to transfer the patients’ registration to the afternoon clinic if available, which would allow relatives with work commitments in the morning to bring them in the afternoon.

*‘Most of our patients are very old, and they are very dependent on their family members to be taken to the centre; otherwise, they don’t come, and their diabetic care is discontinued.’(Dietician, Omani, First model of service delivery)*

*‘Sometimes we transfer our patients to the afternoon clinic for social reasons like transportation. The solution works very well for them and us, as there is always someone to drive them to the clinic in the afternoon but not in the morning.’(Physician, Omani, First and second models of service delivery)*

## Discussion

This study contributes to the process of identifying new challenges in addition to those that already exist in the literature related to diabetes management service delivery at primary health care centres. The identified challenges have evolved into the community and affect the country’s diabetes awareness.

We identified three different models of service delivery; the first model, which was decided by the Ministry of Health in Oman, appears to face numerous challenges. Therefore, some centres have decided to adopt the second model with an appropriate team-based approach to overcome some of these challenges. The third model was found to lack a lot of resources and could not reach the first model level as recommended by the ministry.

The three different models of service delivery at the clinics had different influences on the challenges identified. Most challenges occurred at the regular combined morning clinics and general clinics. The dedicated diabetes management clinics managed to overcome some of these challenges related to diabetes management by opening the clinic in the afternoon. However, other challenges persist and have several implications for diabetes management service delivery, care providers, and patients. Some of the challenges identified require basic interventions; others require complex and long-term solutions. Further discussion of the challenges, their implications and opportunities for improvement will be guided by four perspectives.

### Enhancement of the organisational structure and work stream at diabetes management clinics

This perspective lists basic challenges, such as shared rooms, a shortage of computers, and inefficient support systems. We found that shortages of computers and inefficient support systems have serious implications. The literature in this field is rapidly evolving because of technological advances, increasing access to computer systems in clinical practice, and growing concern about the process and quality of medical care [28], which all needs regular evaluation and updating to improve care providers’ performance and enhance patient outcomes. The study supports regular updates and further evaluations by the ministry.

Patient empowerment is a significant concept in service delivery and can be achieved by enhancing patients’ participation (i.e., preparing patients for visits using assistant-guided patient preparation, empowering group education, group consultations, or automated telephone management), which will eventually lead the service to become more patient-centred [29]. However, shared rooms within the clinic present an obstacle, and it is not clear how handling the patient in one room with two providers could provide patient empowerment. Further, empowerment to the care providers can be achieved through team performance. The care providers presence as a team is essential in diabetes management clinics, as the clinics are the first setting where the disease is identified, diagnosed, treated, and followed-up [30]. We found in this study that care providers in the regular combined morning clinics could not utilize a team-based approach due to the absence of some members or unclear organization of the team. The physicians in the general clinic had to use a single provider approach in most cases and faced inefficient management. The shortages and unavailability of care providers, mainly qualified nurses, as indicated by the physicians, led to unwanted outcomes at the clinic.

The physicians in this study revealed that nurses ill-defined roles and lack of education in diabetes management meant that other providers stopped delegating work to them and did not trust them, which subsequently led to underutilized nurses. They added that those nurses trained in diabetes care work to the best of their abilities; however, these nurses were unfocused and had low self-esteem because they had to cover other clinics in the centre. They stressed that proper selection, education, and training of nurses by the ministry and support in taking an active role in the diabetes teams could improve the service and should be a priority action by the stakeholders of the ministry. The literature supports this fact and underlines that the broadening of nurses’ practice can significantly improve practice, service delivery, and health care systems [31]. The literature adds that proper training and certification improves nurses’ perceived job satisfaction [32], and the role of nurses working in partnership with consumers and in teams that collaborate across disciplines, professions, and sectors is crucial action for achieving sustainable outcomes [33].

Nurses in this study protected themselves by explaining that Omani culture plays a role in the methodology of service provision. They further revealed that their knowledge and skills are appropriate for diabetes management clinics; however, they stressed that most patients prefer to see doctors. The nurses added that their dissatisfaction with higher authorities is a serious issue, which subsequently explained their actions in not further pursuing their careers in diabetes management. The literature confirms this attitude among nurses and mentions that it could lead to other future outcomes including poor nursing career structures and a lack of opportunities for further education [34]. The other providers, including the dieticians and health educators, did not discuss their skills and competencies as weaknesses. However, they found themselves weak in comparison to their colleagues in the private sector in not providing complete diabetes management, although this was not due to their knowledge, but to the absence of diagnostic and education tools. The literature highlighted that the majority of support staff, including nurses, in a clinical setting hold less than a baccalaureate degree and may not have been exposed to formal research courses [35]. Therefore, the physicians are aware of their weaknesses and do not delegate work to them, which consequently leads to patients not trusting them [20].

Workstream and communication incoherence in the diabetes management clinic in this study underlined challenges related to the language barrier of multinationality care providers in the country. The situation is the same globally and has been described as a key need that requires extensive attention and research [36]. The literature in this regard address the use of interpreters as a solution to such challenges; however, primary care providers must be aware of this issue and understand when interpreters are required [37]. Continuity of care thanks to good communication in the clinic is a continuous desire among care providers, as this promotes provider-patient relationships and increases the efficiency of the health care system [38].

### Expanding technical and pharmaceutical support

We found in this study that diabetes management clinics at primary health care centres lack important patient education tools and drugs. The providers concluded that this led to patients’ dissatisfaction and lack of diabetes control, as well as discouraging visits and producing a sense of failure from the care providers’ side. These challenges could be considered basic, but if not addressed, such lack of valuable pharmaceutical support may lead to further complications and poor outcomes in other aspects of care [39].

### Rising patients, families and community obligations

Our results also indicated that the community has a big influence on peoples’ lives and health. Cultural beliefs are very strong; the trust between the people is stronger than the trust in care providers. The providers revealed that patients are very open to traditional healing management or to attending private clinics for second opinions. These practices, as revealed by the providers, decrease treatment compliance and increase the number of defaulters in the public primary health care sector. We found that the patients’ disregard for the appointment system has many serious consequences that require extensive communications and strategies by the care providers. This issue was considered a complex challenge because it involves patient behaviour and causes considerable distress among providers. However, all expressed that it could be tackled with communication, not only at the patient level but also at the family and community levels. The literature supports our findings and shows that anyone who has treated patients knows that the adoption of a new behaviour, the cessation of an unwanted behaviour, or the commitment to adhere to medical advice is central to effective medical care [40, 41]. Also, the care providers achieving behaviour change in their patients through various methods of patient interaction is a complex subject [42].

### Building health and diabetes awareness in Oman

A sweet diet and a sedentary lifestyle are not uncommon practices among patients. Our study exposed these findings and supports existing findings in the literature [43]. Our study indicates that knowledge about health and diabetes might be limited among patients and in the community. Providing community health workers with tools to support their work, such as training and supportive supervision, might increase awareness and responsiveness to different diseases. The literature supports such actions and has found them to be essential in the community [44], and we found from the study these actions are essential but require a lot of community support. According to care providers, the denial of diabetes and low independence in its management leads to low compliance with medications, insulin, and healthy lifestyles. The literature states that such challenges are not easy to address and require extensive and effective team-based approaches at the clinics, as patients self-management and its support are considered the core outcome of a successful team-based clinic [45]. The developers of health services in the literature emphasise that offering a framework for the members of the team with specific tasks could help to provide appropriate health services for people with long-term conditions [46, 47]. In public health centres, the care providers admitted that patient self-management and its support is an issue but can be improved by providing proper diabetes management teams [20]. We found that media can also play a significant role. However, the challenge and the solution should be acknowledged at the health centre level first, with further supportive and advisory work from the media for the prevention of diabetes and other diseases in schools, colleges, companies, and all institutes of the country.

### Methodological considerations and limitations

The study captured range of challenges related to diabetes management clinic at primary health care centres and the community. The centres were of mixed criteria. The sample of patients was selected from the daily appointment lists in the clinics, so the age and duration of disease were representative of actual diabetic patients in Oman. The study did not include gender comparison; however, the low number of observed consultations with male patients can be explained by the low numbers of male diabetic patients registered at the centres compared to females [48]. It is not known whether the same results would have been noticed if the gender distribution had been equal. The sample of care providers represented all three models of service delivery available in public primary health care centres in Muscat. The low contribution from male care providers was also due to the low numbers of male care providers compared to females in primary health care [20].

The observational part of the study allowed the researcher to experience the diabetes service provision in an Omani context and the interviews further explained the collected data. The observations were also used as a basis for the interviews, which gave a bigger scope and better understanding of the challenges.

The data in this study was collected by the first author, who is an Omani physician at the Ministry of Health (insider), but not at primary health care centres and not positioned in diabetes management clinics (outsider); this gave dual roles and provided positive and negative inputs for the study.

The insider part gave a positive credit for being part of the system having a pre-understanding of the situation. At the same time, this pre-understanding was handled with care by giving all care providers the same chance to discuss the challenges and opportunities for improvement from the same perspective. On the other hand, the outsider aspect gave positive credit for not being judgmental of the challenges identified and not justifying their implications.

A negative aspect is that the first author is part of the hierarchical system at the Ministry of Health, where physicians are placed at the top of the system. This empowerment allowed the researcher to be at the same level as the physicians and allowed them to be more expressive about the challenges compared to other providers, and more blameful towards them. Although the interviews were performed professionally, with maximum research abilities, it was a challenge to interview the other providers due to their cultural understanding that they are lower in status than physicians, and the challenges mentioned were in the context of the clinics’ structure and tools only, with no blaming actions towards the physicians.

The interviews generated numerous statements from the physicians. However, appropriate space was given to other providers to balance all respondents perceptions, and other providers views and insights were expanded in the result and discussion sections.

The data on patients from the interviews came from care providers only. Patients’ perceptions of the challenges and improvements at diabetes management clinics are also very important, and future studies and papers in this area will be addressed.

Researchers who conducted this study are from different contexts, and this provides additional positive credits to the study, as what is taken for granted by some researchers might not be well known by others. Regarding data analysis, the first author performed the first set of coding and the third author, who does not work within the system in Oman and was considered an external researcher reinforced this with further qualitative data analysis and interpretation. Furthermore, all the authors have different experiences in the field of diabetes, and further discussion was carried out through regular peer de-brief sessions.

The study included care providers from five public primary health care centres in Muscat. Therefore, the findings might not apply to the other centres in Muscat or to other regions of Oman. However, the providers, the patients, and the structure of the centres are almost the same throughout Oman, and the five centres can be considered a representative sample for public primary care.

## Conclusions

Diabetes management clinics at primary care level face several challenges, with varying degrees of severity and further community involvement. The most crucial challenge, which requires serious and rapid intervention, is the identification of nurse roles and tasks with team-based diabetes management. According to physicians, the challenge comprises weak diabetes knowledge, skills, and competencies among nurses. However, the nurses indicated that the main issue is the lack of support from higher authorities in retrieving knowledge about team-based diabetes management in theory and practice. Many other challenges exist, and opportunities for improvement are available. However, this might take different forms and timescales and requires extensive involvement, shared responsibilities, and implications from the government, health centres, and community. The challenges perceived in the study were related to providers’ perceptions, and the perception of patients and people in the community is unknown. It is also unknown what challenges are faced in the private primary care sector. Therefore, further studies that involve patients’ perceptions or community perception might be suggested in addition to studies involving the private sector.

## Abbreviations

GDP: Gross domestic product
IT: Information technology

## References

[1] Wagner EH, Austin BT, Davis C, Hindmarsh M, Schaefer J, Bonomi A (2001). Improving chronic illness care: translating evidence into action. Health Aff.

[2] Al-Mandhari A, Al-Adawi S, Al-Zakwani I, Dorvlo A, Al-Shafaee M (2013). Reasons for consultation among patients attending primary healthcare centres in Oman. Sultan Qaboos Univ Med J.

[3] Van Lerberghe W. The world health report 2008: primary health care: now more than ever: World Health Organization; 2008.

[4] Hanan A-A, Roland M (2005). Quality of primary health care in Saudi Arabia: a comprehensive review. Int J Qual Health Care.

[5] Margolis SA, Carter T, Dunn EV, Reed RL (2003). Primary health care services for the aged in the United Arab Emirates: a comparison of two models of care. Asia Pac Fam Med.

[6] Lillemoen L, Pedersen R (2013). Ethical challenges and how to develop ethics support in primary health care. Nurs Ethics.

[7] Almalki M, FitzGerald G, Clark M (2011). Health care system in Saudi Arabia: an overview/Aperçu du système de santé en Arabie saoudite. East Mediterr Health J.

[8] Campbell SM, Roland MO, Buetow SA (2000). Defining quality of care. Soc Sci Med.

[9] Epping-Jordan J, Pruitt S, Bengoa R, Wagner E (2004). Improving the quality of health care for chronic conditions. Qual Safety Health Care.

[10] Chen L, Magliano DJ, Zimmet PZ (2012). The worldwide epidemiology of type 2 diabetes mellitus—present and future perspectives. Nat Rev Endocrinol.

[11] Zimmet PZ, Magliano DJ, Herman WH, Shaw JE (2014). Diabetes: a 21st century challenge. Lancet Diab Endocrinol.

[12] Badran M, Laher I. Type II diabetes mellitus in Arabic-speaking countries. Int J Endocrinol. 2012;2012. 10.1155/2012/902873 PMC3407623 22851968

[13] Al-Shookri A, Khor GL, Chan YM, Loke SC, Al-Maskari M. Type 2 diabetes in the Sultanate of Oman. Malays J Nutr. 2011;17(1). 22135872

[14] Wens J, Vermeire E, Van Royen P, Sabbe B, Denekens J (2005). GPs' perspectives of type 2 diabetes patients' adherence to treatment: a qualitative analysis of barriers and solutions. BMC Fam Pract.

[15] Lawton J, Peel E, Parry O, Araoz G, Douglas M (2005). Lay perceptions of type 2 diabetes in Scotland: bringing health services back in. Soc Sci Med.

[16] Van den Arend I, Stolk R, Krans H, Grobbee D, Schrijvers A (2000). Management of type 2 diabetes: a challenge for patient and physician. Patient Educ Couns.

[17] Abdulhadi N, Al-Shafaee MA, Östenson C-G, Vernby Å, Wahlström R (2006). Quality of interaction between primary health-care providers and patients with type 2 diabetes in Muscat, Oman: an observational study. BMC Fam Pract.

[18] Abdulhadi N, Al Shafaee M, Freudenthal S, Östenson C-G, Wahlström R (2007). Patient-provider interaction from the perspectives of type 2 diabetes patients in Muscat, Oman: a qualitative study. BMC Health Serv Res.

[19] Abdulhadi NMN, Al-Shafaee MA, Wahlström R, Hjelm K (2013). Doctors’ and nurses’ views on patient care for type 2 diabetes: an interview study in primary health care in Oman. Primary Health Care Res Dev.

[20] Al-Alawi K, Johansson H, Al Mandhari A, Norberg M. Are the resources adoptive for conducting team-based diabetes management clinics? An explorative study at primary health care centers in Muscat, Oman. Primary Health Care Res Dev. 2018:1–28. 10.1017/S1463423618000282 PMC6476396 29737963

[21] Snoek FJ (2002). Breaking the barriers to optimal glycaemic control--what physicians need to know from patients' perspectives. International journal of clinical practice Supplement.

[22] Oman Population [http://worldpopulationreview.com/countries/oman-population/].

[23] Oman Demographic Profile. In: Fact Book. 2016.

[24] Oman: WHO statistical profile [http://www.who.int/countries/omn/en/].

[25] MOH: Ministry of Health Annual Health Report. In*.* Directorate General of Planning, Department of Information and Statistics; 2016.

[26] McCambridge J, Witton J, Elbourne DR (2014). Systematic review of the Hawthorne effect: new concepts are needed to study research participation effects. J Clin Epidemiol.

[27] Vaismoradi M, Turunen H, Bondas T (2013). Content analysis and thematic analysis: implications for conducting a qualitative descriptive study. Nurs Health Sci.

[28] Garg AX, Adhikari NJ, McDonald H (2005). Effects of computerized clinical decision support systems on practitioner performance and patient outcomes: a systematic review. JAMA.

[29] van Dam HA, van der Horst F, van den Borne B, Ryckman R, Crebolder H (2003). Provider–patient interaction in diabetes care: effects on patient self-care and outcomes: a systematic review. Patient Educ Couns.

[30] Atun R (2004). What are the advantages and disadvantages of restructuring a health care system to be more focused on primary care services.

[31] Fairman JA, Rowe JW, Hassmiller S, Shalala DE (2011). Broadening the scope of nursing practice. N Engl J Med.

[32] Wyatt J, Harrison M (2010). Certified pediatric nurses’ perceptions of job satisfaction. Pediatr Nurs.

[33] Sheridan N, Finlayson M, Jones M (2009). Guest editorial: position statement: primary health care nursing. J Prim Health Care.

[34] Buchan J (2002). Global nursing shortages: are often a symptom of wider health system or societal ailments. BMJ: British Medical Journal.

[35] Marquis BL, Huston CJ. Leadership roles and management functions in nursing: theory and application: Lippincott Williams & Wilkins; 2009.

[36] Jacobs E, Chen AH, Karliner LS, AGGER-GUPTA N, Mutha S (2006). The need for more research on language barriers in health care: a proposed research agenda. The Milbank Quarterly.

[37] Gray B, Hilder J, Stubbe M (2012). How to use interpreters in general practice: the development of a New Zealand toolkit. J Prim Health Care.

[38] Lambrew JM, Defriese GH, Carey TS, Ricketts TC, Biddle AK (1996). The effects of having a regular doctor on access to primary care. Med Care.

[39] WHO: Primary health care: 25 years after ALMA-ATA. 2003.

[40] Needham I, Abderhalden C, Halfens RJ, Fischer JE, Dassen T (2005). Non-somatic effects of patient aggression on nurses: a systematic review. J Adv Nurs.

[41] Bailey RD, Clarke M. Stress and coping in nursing: Springer; 2013.

[42] Rollnick S, Miller WR, Butler CC, Aloia MS: Motivational interviewing in health care: helping patients change behavior. In: Taylor & Francis; 2008.

[43] Kilani H, Al-Hazzaa H, Waly MI, Musaiger A (2013). Lifestyle habits: diet, physical activity and sleep duration among Omani adolescents. Sultan Qaboos Univ Med J.

[44] Jaskiewicz W, Tulenko K (2012). Increasing community health worker productivity and effectiveness: a review of the influence of the work environment. Hum Resour Health.

[45] Bodenheimer T, Ghorob A, Willard-Grace R, Grumbach K (2014). The 10 building blocks of high-performing primary care. Annals Family Med.

[46] Pearce G, Parke HL, Pinnock H, Epiphaniou E, Bourne CL, Sheikh A, Taylor SJ (2016). The PRISMS taxonomy of self-management support: derivation of a novel taxonomy and initial testing of its utility. J Health Serv Res Policy.

[47] Sheridan N, Kenealy T, Kuluski K, McKillop A, Parsons J, Wong-Cornall C. Are patient and carer experiences mirrored in the practice reviews of self-management support (PRISMS) provider taxonomy? Int J Integr Care. 2017;17(2). 10.5334/ijic.2483 PMC5624079 28970749

[48] Al-Lawati JA, Tuomilehto J (2007). Diabetes risk score in Oman: a tool to identify prevalent type 2 diabetes among Arabs of the Middle East. Diabetes Res Clin Pract.

